# Combined effects of soil 3D spatial heterogeneity and biotic spatial heterogeneity (plant clumping) on ecosystem processes in grasslands

**DOI:** 10.1002/ece3.10604

**Published:** 2023-10-24

**Authors:** O. Vindušková, G. Deckmyn, M. Bortier, H. J. De Boeck, Y. Liu, I. Nijs

**Affiliations:** ^1^ Plants and Ecosystems (PLECO) Department of Biology, University of Antwerp Wilrijk Belgium; ^2^ Institute for Environmental Studies Faculty of Science, Charles University Prague Czech Republic; ^3^ School of Ecology and Environmental Sciences Yunnan University Kunming China; ^4^ State Key Laboratory of Herbage Improvement and Grassland Agro‐ecosystems, Key Laboratory of Grassland Livestock Industry Innovation, Ministry of Agriculture and Rural Affairs College of Pastoral Agriculture Science and Technology, Lanzhou University Lanzhou China

**Keywords:** competition, niche, patch size, plant biomass, resource uptake, soil variability

## Abstract

Soil heterogeneity has been shown to enhance plant diversity, but its effect on grassland productivity is less clear. Even less is known about the effect of plant clumping (intraspecific aggregation) and its potential interaction with soil heterogeneity. The combined effects of soil 3D spatial heterogeneity and species clumping were experimentally studied in grassland mesocosms consisting of four grassland species. These species were planted in three patterns (i.e. completely mixed, clumped by 9 or 36 individuals of the same species) on soils with heterogeneous cells of alternating nutrient‐poor and rich soil differing in size from 0 (mixed soil) to 12, 24, and 48 cm (complete poor or rich mesocosm). Moderate soil cell sizes (12–24 cm) consistently increased whole‐mesocosm aboveground productivity by more than 20%, which mainly originated from the increased growth of the plants growing on the poor soil cells. In contrast, total mesocosm productivity was not affected by species clumping although there were some species‐specific effects, both of clumping and of the interaction of clumping with soil heterogeneity. Our results show that intermediate soil heterogeneity promotes productivity. Clumping can improve the growth of inferior species, thus promoting coexistence, without affecting overall productivity. We found no interaction effect of clumping and soil heterogeneity on productivity at the community level and some minor species‐specific effects.

## Graphical abstract

The combined effects of soil 3D spatial heterogeneity and species clumping were experimentally studied in grassland mesocosms consisting of four grassland species. Our results show that intermediate soil heterogeneity promotes productivity. Clumping can improve the growth of inferior species, thus promoting coexistence, without affecting overall productivity.

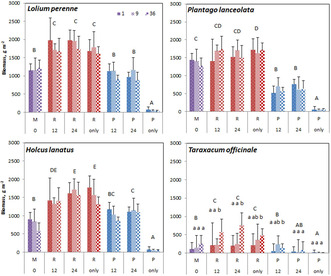

## 1 INTRODUCTION

In nature, soil can be variable even at small scales surrounding individual plants, with significant differences in pH, nutrient availability, and soil density occurring in samples from within centimeters of each other (Farley & Fitter, 1999; Jackson & Caldwell, 1993), often as a result of soil fauna activity or animal grazing and excreta. The importance of this spatial heterogeneity on ecosystem functioning has been intensively studied at different levels and in very contrasting settings. Several studies have shown that understanding the effects of grazing patterns, excretion patterns, and soil heterogeneity on grassland functioning is critical to predict field‐scale processes and the implications on ecosystem service provision (Berisha & Geci, 2023; Bloor & Pottier, 2014; Xi et al., 2014).

Environmental heterogeneity usually promotes species coexistence and diversity (Helbach et al., 2022; Lundholm, 2009; Stein et al., 2014) and the same is true for soil heterogeneity (Stover & Henry, 2018). For example, the homogenization of soils by tillage which reduces heterogeneity consequently reduces diversity (Stover & Henry, 2020a). Theoretically, environmental heterogeneity promotes niche differentiation, where species with little niche overlap can coexist (Chesson, 2003; Tilman & Pacala, 1993). Stover and Henry (2018) hypothesized that in addition to the main niches created by soil patches of different properties themselves, additional niche spaces may open at the “micro‐edges” between patches, in a small‐scale analogy with the ecotone concept. Kowalski and Henry (2020) confirmed this hypothesis by showing that, depending on the substrates used, the border between more nutrient‐rich and nutrient‐poor cells can become a separate niche and therefore increase potential diversity and resource acquisition. Questad and Foster (2008) also showed in a manipulation experiment that species coexistence is related to species sorting along heterogeneous niches. Recently, a higher number of soil patch types (4 vs. 2) has been experimentally shown to increase diversity, further supporting the niche theory (Xue et al., 2021).

Since species diversity in turn determines ecosystem functioning (Hooper et al., 2012), especially productivity, relationships between environmental heterogeneity and ecosystem functioning and productivity can be expected. Theoretically, a diverse community should be more productive because more of the available resources are used when species with different traits grow together (Price et al., 2014). Many studies have focused on the link between soil heterogeneity and species diversity as a driver for ecosystem functioning in terms of productivity and C‐sequestration (Cong et al., 2014; De Deyn et al., 2011; Schaub et al., 2020) though these studies show contrasting results. For example, in a study by Xue et al. (2016) species richness increased productivity but soil heterogeneity did not, nor did these factors interact. Gundale et al. (2011) likewise found little effect of soil heterogeneity on the diversity‐productivity relationship.

Tylianakis et al. (2008) used structural equation modeling to show that the slope of the relationship between diversity and productivity increases when limiting resources are spatially heterogeneous. This confirms the dependence of the diversity/productivity relationship on the overall productivity, as also stated by Yang et al. (2015).

Other studies have avoided the complexity of the interaction between heterogeneity, diversity, and productivity, by focusing on the responses of monocultures to variation in soil heterogeneity (Wijesinghe & Hutchings, 1997, 1999). A key mechanism found in several experiments is that plants react to heterogeneous soils by enhancing their root/shoot ratio (Hutchings & John, 2004) or by increased nutrient uptake rate per unit root surface area, resulting in a better acquisition of the available soil nutrients and water, though these responses are very species‐dependent (Robinson, 1994). The importance of root plasticity for such processes has been demonstrated by several authors and can explain some of the differential responses of species to soil heterogeneity (Hutchings et al., 2003).

In the experiments on the effects of soil spatial heterogeneity, another form of heterogeneity was to our knowledge seldom included: species clumping or intraspecific aggregation. In contrast to the abiotic heterogeneity of the soil, this type of heterogeneity is biotic. It can be caused by seeds falling close to parent plants or by clonal growth, but also by abiotic heterogeneity when monospecific clumps track and thus mirror the patchiness of suitable micro‐environments (Suzuki et al., 2005). Species clumping has important effects on ecosystem functioning. In experiments with dominant and inferior competitors planted or sown together, a dominant species generally performs better next to an inferior species because it acquires more resources (nutrients, water, and/or light) compared to a setting where it is grown in monoculture and therefore undergoes intraspecific competition (Stoll & Prati, 2001). The reverse is true for the inferior competitor, which will experience less competition in local monocultures (i.e. clumps). Xue et al. (2018) in an experiment with two species found no evidence that intraspecific aggregation alters competitive interactions, but soil heterogeneity did affect the relative yield of the species. Damgaard (2010) in a field study in North‐European dune grassland, found no additional support for the hypothesis that reduced interspecific competition due to intraspecific aggregation is important for maintaining a species‐rich flora. Zhang et al. (2014) observed a positive correlation between the productivity of species‐rich grasslands and the frequency of interspecific interactions, which decreases with increased species clumping. This seems to confirm at the plant neighborhood scale the paradigm that diversity begets productivity. Also for biotic heterogeneity, the three factors of heterogeneity, diversity, and productivity thus seem linked.

In nature, exploring the role of heterogeneity is hampered by co‐varying factors (nutrients, soil depth, etc.) which render it problematic to clearly link cause and effect. In this study, we conducted an outdoor mesocosm experiment to compare the responses of experimental plant communities of four grassland species concerning productivity, resource use efficiency, and intra‐ and interspecific competition when growing on soils of different three‐dimensional (3D) spatial heterogeneity, in different clump sizes, or the combination of both in a fully crossed design. The experimental mesocosms were constructed using a recently developed technique (Liu, De Boeck, et al., 2017) by filling neighboring cells with alternating nutrient‐rich and nutrient‐poor soil. Plants were then planted in clumps of different sizes. Gradients of decreasing soil and biotic heterogeneity were created by increasing the soil cell size (0,12, 24, and 48 cm) and plant clump size (1, 9, and 36), respectively.

We hypothesize that productivity is enhanced at intermediate soil cell sizes because three niches are available to the plants: the center of the resource‐poor patches, the edge between patches, and the center of the resource‐rich patches. We expect species clumping to benefit the competitively inferior species as these are protected within clumps against the dominant species, which could lead to a reduced overall productivity at the community level if the clump size is big (i.e. interspecific interactions become insignificant). At intermediate clumping, the result will depend on whether the improved resource uptake at the patch edges can compensate for this reduction of productivity in the patches of suppressed species.

## 2 MATERIALS AND METHODS

### 2.1 Experimental setup

The experiment was conducted at the University of Antwerp, Belgium (51°09′41″ N, 04°24′29″ E). The local climate is characterized by mild winters (average temperature in January is 3.4°C) and cool summers (average temperature in July is 18.5°C), with an average annual temperature of 10.6°C and annual rainfall of 848 mm, equally distributed throughout the year (Royal Meteorological Institute of Belgium).

In spring 2017, five treatments of 3‐dimensional soil heterogeneity were created layer by layer in wooden boxes (“mesocosms”) of 48 × 48 × 48 cm through filling two types of soil: nutrient‐poor sandy soil (“poor soil,” 80% sand and 20% potting soil) and nutrient‐rich soil with high content of organic matter (“rich soil,” 80% potting soil and 20% sand; see Table 1) alternatingly in both the horizontal and vertical direction (for further details see Liu, Bortier, et al., 2017). The nutrient values of the poor and rich soil are close to the existing values of rich and poor soils in the field (Liu, Bortier, et al., 2017).

**TABLE 1:** Soil characteristics.

Soil type	pH KCl	C (%)	NaCl (mg/L)	NO_3_‐N (kg ha^−1^)	NH_4_^+^‐N (kg ha^−1^)	P_2_O_5_ (mg/L)	K_2_O (mg/L)	MgO (mg/L)	CaO (mg/L)	Na_2_O (mg/L)
Nutrient‐poor	5.1	0.1	489	66	11	70	74	25	706	18
Nutrient‐rich	6.3	1.9	2176	380	19	1038	736	601	2955	132

**FIGURE 1:**
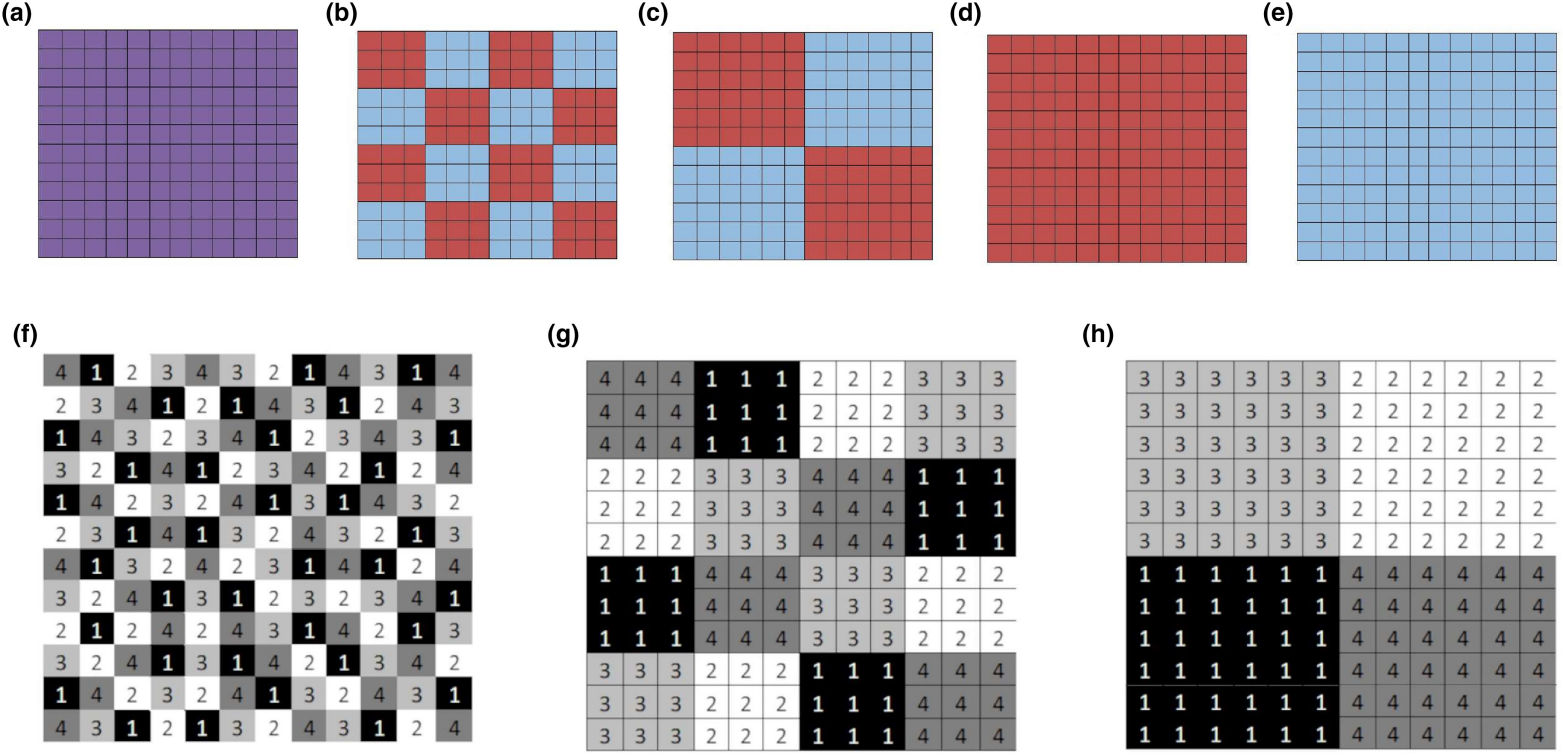
Mesocosms of 48 × 48 cm depicted in squares of 4 × 4 cm. Top shows five levels of soil heterogeneity at the mesocosm scale, that is, mixed soil (cell size 0 cm) (a), cell sizes 12 cm (b), 24 cm (c), and 48 cm: pure rich (d) or poor soil (e). Bottom (f–h) shows three levels of species clumping design of four species in clumps of 1, 9, or 36 individuals, respectively.

Mesocosms with cell sizes 24 and 12 cm were filled with poor and rich soil in an alternating fashion as described above (Figure 1b,c). Mesocosms with cell size 0 cm were filled with a mixture of the two substrates (Figure 1a), with poor and rich soil alternating at short range. Here, the exact distance between rich and poor patches is unknown but <1 cm. We labeled this treatment M (mixed) for simplicity. Finally, mesocosms with a cell size of 48 cm were filled with either poor or rich soil, creating two types of mesocosms with 48 cm soil cell size (P and R, respectively, Figure 1d,e). In this experiment, qualitative heterogeneity (texture, nutrients, moisture, pH, etc.) was constant since the same two substrates were applied, while only configurational heterogeneity (the size and distribution of the patches) was modified by varying cell size from 0 to 24 cm. Note that on the pure R and P soils (48 cm), the distance between resource‐rich and resource‐poor patches can in this case be considered as infinite because there is no adjacent patch with a different substrate (from where roots might grow in, for example), therefore in our analyses we always show the rich and poor mesocosm of the 48 cm separately, but the average of these data can be used as comparison for soil heterogeneity with an infinite cell size.

Seeds of four co‐occurring grassland species, very common in Belgium and most of Europe, were sown in trays on 29 March 2017, and planted in a specific design into the mesocosms at the end of April (two‐leaf stage): two perennial grasses (*Lolium perenne* L. and *Holcus lanatus* L.), and two common grassland dicots (*Plantago lanceolata* L. and *Taraxacum officinale*). Each mesocosm contained all four species. They were either completely mixed (no conspecific neighbor), clumped per 9 (3 × 3), or per 36 (6 × 6) individuals of the same species (Figure 1f–h). The individuals were planted at a density of 36 per species per mesocosm, at 4 cm intervals. At the largest clumping level, each species therefore covered one‐quarter of the mesocosm (Figure 1h).

In total, five replicates were constructed of each of the five treatments of soil heterogeneity (four cell sizes, but for the largest cell size of 48 cm both R and P soil mesocosms), crossed with the three clumping levels. For the clumping per 36 individuals on the 24 cm patches, five additional mesocosms were constructed because with this pattern two species are on rich soil and two on poor soil, yielding 80 mesocosms in total. The pattern of the species clumps over the soil surface was carefully designed to have an equal number of the different species interactions in each replicate. Moreover, the five replicates of the species clumps were not identical with respect to species positions to ensure all interspecific interactions were equally present in the whole experiment (Figure 1). New seedlings emerging during the experiment were removed. The mesocosms were exposed to ambient weather conditions and irrigated to field capacity when soil water content (SWC) was below 2/3 of field capacity (see measurements below). The experiment lasted for 6 months (until the beginning of October 2017).

### 2.2 Measurements

Measurements focused on the influences of soil heterogeneity and clumping on plant productivity and the acquisition of the most important underlying resources of plant biomass production (light, nutrients, and water). Biomass was harvested by species and for the rich and poor patches separately from each mesocosm, by cutting 2–3 cm above the ground at the end of June and the first week of October 2017 (the end of the experiment). The material was dried for 48 h at 70°C and weighed. Photosynthetically active radiation (PAR) was measured with a small custom‐made sensor on overcast dry days on 24–26 July 2017, on 16 sampling points (middle of each 12 × 12 cm patch) per mesocosm (80 × 16 = 1280 measurements in total), above and below the canopy. From the fraction of incident PAR absorbed by the canopy, we assessed how the soil heterogeneity and clumping treatment influenced the capacity of the communities to absorb radiation. Nitrogen acquisition was assessed indirectly via the chlorophyll and nitrogen status of the plants. This was determined by measuring the chlorophyll content and the Nitrogen Balance Index (NBI), a measure for N‐deficiency, with a Dualex optical leaf clip meter (Force‐A, Orsay, France). In this instrument, chlorophyll content is calculated from leaf transmission in near‐infrared and infrared (in μg/cm), and the NBI (in relative units 0–100) from the ratio of the chlorophyll and flavonol content (both are decreased under N deficiency). Measurements were performed twice, at the first and final harvest, on one youngest fully expanded leaf per species from both a rich, poor, or mixed soil patch in each mesocosm. We collected 420 measurements from 4 species × 7 types of patches within soil heterogeneity treatments (M0, R12, R24, pure R, P12, P24, pure R) × 3 clumping levels × 5 replicates per time point. This allowed us to determine how the soil heterogeneity and clumping treatment influenced the capacity of the communities to take up nutrients.

Soil water content was monitored with a profile probe (PR2, Delta‐T Devices Ltd.) placed in access tubes in the middle of all 80 mesocosms. Data were registered at four soil depths (average depth 5.5, 16.4, 27.3, and 38.2 cm) from which the SWC was calculated in three layers of equal thickness: top (5.5–16.4 cm), middle (16.4–27.3 cm) and bottom layer (27.3–38.2 cm), in each case by averaging the top and bottom value of the layer. The probe was calibrated based on mass loss over time before the actual experiment started. SWC was monitored weekly to apply irrigation when soil water dropped (see above). To calculate the water uptake rate, a period was chosen when the soils started fully saturated, but precipitation was then absent for 2–3 consecutive days. This happened from August 23 to 25 (second growing period). From the hourly SWC over the daylight hours, the period during which the slope was linear (middle of the day) was selected and the rate of SWC decline (slope of the reductions in SWC) was calculated. This allowed us to assess how the soil heterogeneity and clumping treatment influenced the capacity of the communities to take up water.

Besides these estimates of biomass and resource acquisition, the mortality of the plants was registered at the final harvest as the number of living and dead plants per mesocosm compared to the initial number planted, per soil type, and species. During the experiment, new seedlings emerging were removed.

### 2.3 Statistics

To analyze the effect of soil heterogeneity treatment (M‐12‐24‐P‐R) and clumping (1‐9‐36) and their interaction we used all data available per mesocosm (biomass, PAR absorption, water uptake), performed a Shapiro Wilk normality test and an ANOVA using R 3.3.3. (equation: *y* ~ treatment * clumping). For water uptake, data from all layers were analyzed first using a single ANOVA model (*y* ~ treatment * clumping * layer) and then per layer as described above. Assumptions for using the ANOVA model were assessed visually (homogeneity of variances, normality of residuals) and using the Shapiro–Wilk test (normality of residuals). When there were significant effects, a Tukey test of multiple comparisons of means was performed to find which values were statistically different (95% family‐wise confidence level).

In addition, to study the effects at species and patch level on biomass, chlorophyll, NBI, and mortality, the effects of species (four species), soil patch type within soil heterogeneity treatments (M0, R12, R24, pure R, P12, P24, pure R), and clumping (1, 9, 3) were analyzed with linear mixed‐effect (LME) model with mesocosm as random effect (Satterthwaite's method, *y* ~ species * treatment * clumping + (1|mesocosm)). The same analysis without the species factor was carried out on PAR interception data. Assumptions for using the LME model were assessed visually (homogeneity of variances, normality of residuals) and using the Shapiro–Wilk test (normality of residuals). The package and function emmeans (1.6.3) were used to compute estimated marginal means for multiple comparisons.

## 3 RESULTS

### 3.1 Productivity at mesocosm scale

**FIGURE 2:**
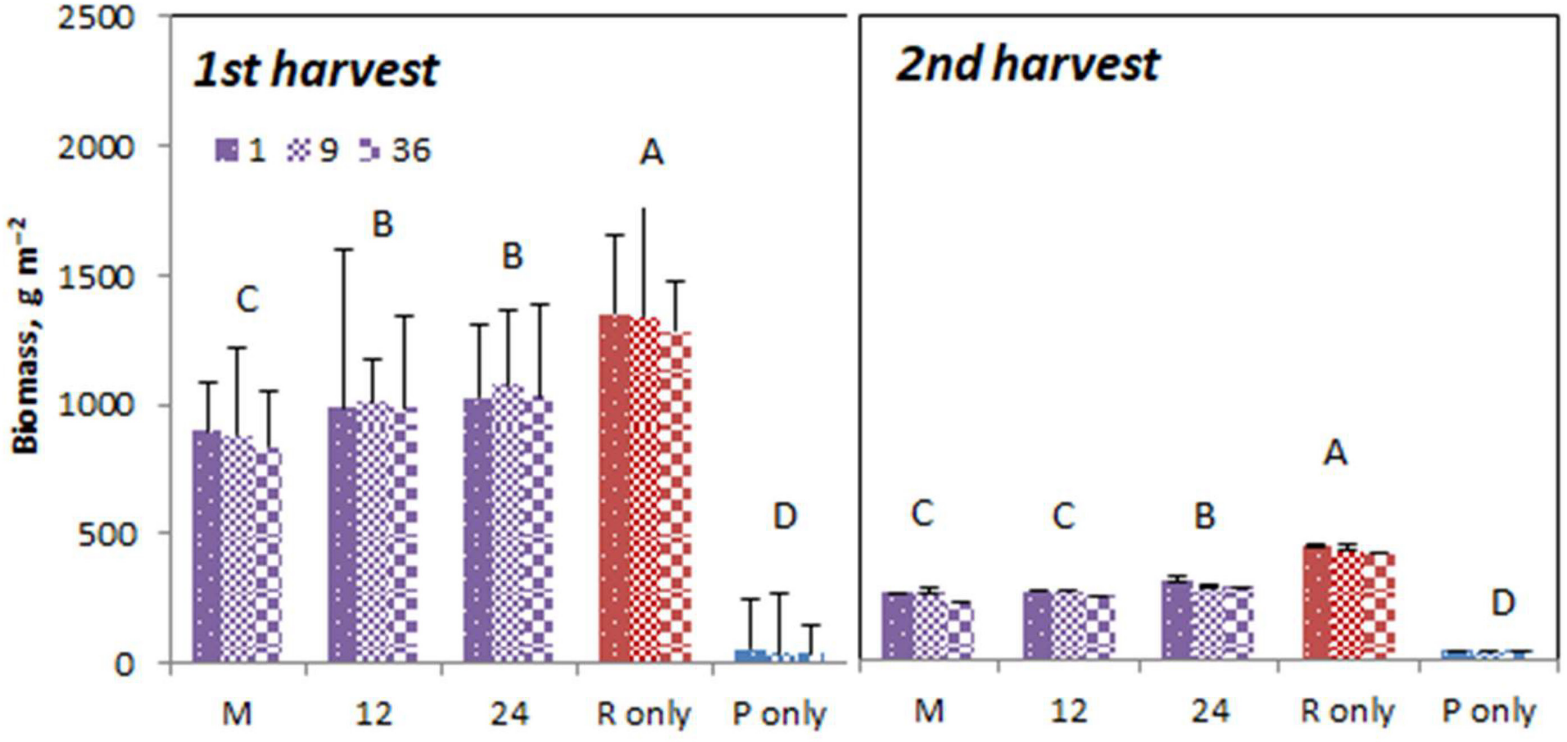
Total aboveground biomass per mesocosm ± SE in function of soil heterogeneity treatment in June and October 2017. Each mesocosm consisted of four grassland species, grown at different clumping sizes from separate individual plants (1), over 9 to 36 of the same species. Significant effects of the soil heterogeneity treatment are indicated.

Total mesocosm yield was much higher at the first harvest (start of summer) compared to the autumn harvest (Figure 2). Not surprisingly, the highest and lowest values were found on pure rich and poor soils, respectively, but for soils of intermediate fertility (purple bars) the values were higher at a soil cell size of 12 and 24 cm (first harvest) or 24 cm (second harvest) compared to the mixed soil (Figure 2; Table 2). Interestingly, the productivity on the mixed soil was higher than the average of the pure rich and poor soil (Figure 2). Species clumping had no effect on total biomass production at the mesocosm level, neither alone nor in interaction with soil heterogeneity (Figure 2; Table 2).

**TABLE 2:** ANOVA results were performed on mesocosms with three clumping levels (1‐3‐9) and five soil heterogeneity (SH) treatments (M‐12‐24‐R‐P).

Parameter	Factor	df	*F*	*p*
Biomass 1	SH	4	450.2	***
	Clumping	2	1.11	
	SH:Clump	8	0.28	
Biomass 2	SH	4	317	***
	Clumping	2	2.59	
	SH:Clump	8	0.64	
PAR interception	SH	4	246.9	***
	Clumping	2	2.84	
	SH:Clump	8	1.06	
Water use	SH	4	30.38	***
	Clumping	2	0.27	
	Layer	2	45.33	***
	SH:Clump	8	1.32	
	SH:Layer	8	2.47	*
	Layer:Clump	4	0.79	
	SH:Clump:Layer	16	0.50	

*Note*: Biomass results at the first harvest (end of July) and second harvest (end of October), PAR interception, and water use. Degrees of freedom (df) and *F*‐values are reported. Asterisks denote *p*‐values <.001 (***) and .05 (*).

### 3.2 PAR interception at mesocosm scale

**FIGURE 3:**
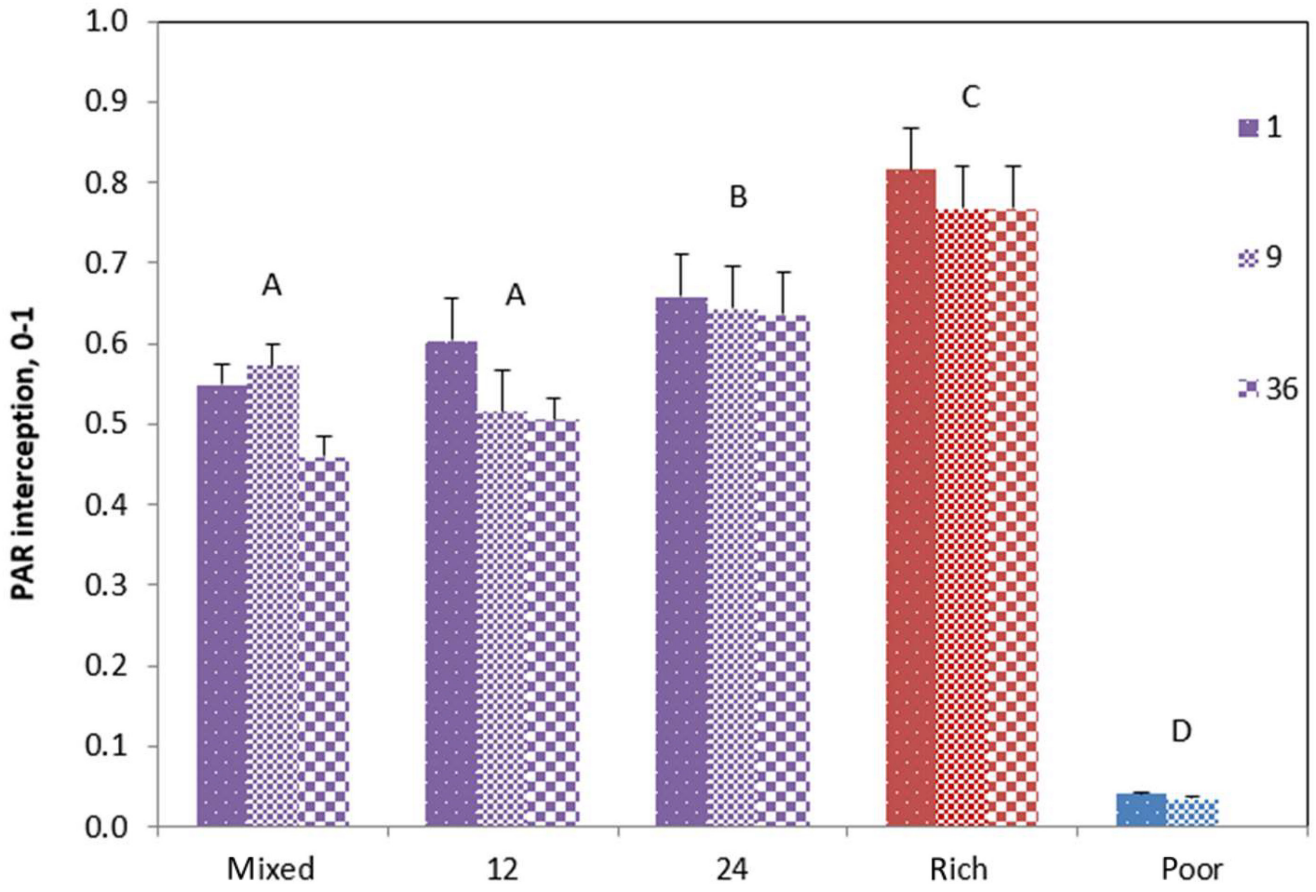
PAR interception ± SE of mesocosms in function of soil heterogeneity treatment for different clumping levels (1,9,36). Significant differences between the treatments are indicated with capitals.

Soil cell size also affected PAR interception (Figure 3; Table 2). The values for the 24 cm soil cell size were higher compared to the 0 (mixed) and 12 cm treatments. Again, the pure rich and poor mesocosms had the highest and lowest values, respectively. Clumping had no overall effect on PAR interception at the mesocosm scale, and the interaction between clumping and soil cell size was also not significant (Table 2).

### 3.3 Soil water use at mesocosm scale

**FIGURE 4:**
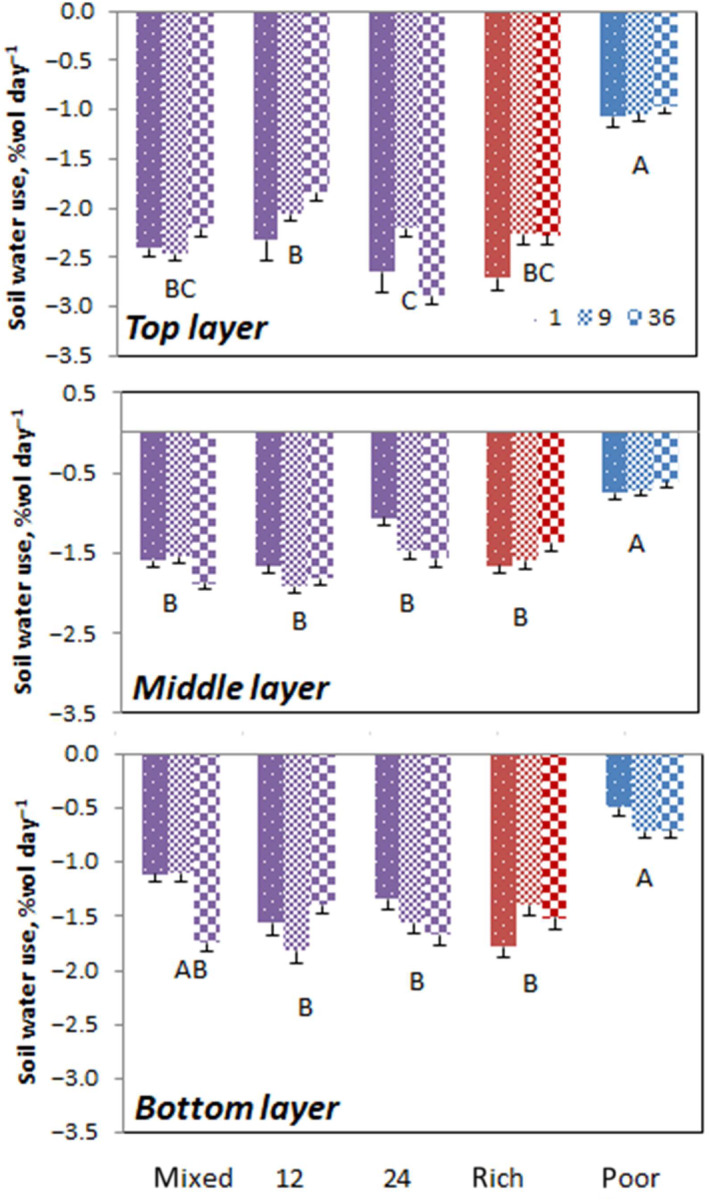
Soil water use in reduction of soil volumetric % water content per day ± SE, at three soil layers in mesocosms in function of soil heterogeneity treatment for different clumping levels (1,9,36). Significant differences between the treatments are indicated with capitals.

The highest amount of soil water was extracted from the top layer (Figure 4; Table 2). Soil water use was also affected by soil heterogeneity, the effect of which differed among layers, as indicated by the significant interaction (Table 2). In the top layer, more water was used in the 24 cm cell size mesocosms than the 12 cm cell size mesocosms (Figure 4). Poor soil mesocosms showed the lowest water use, which was true also for the second and third layers. In these layers, soil cell size did not affect water use. Clumping or its interaction with soil heterogeneity did not affect soil water use in any layer.

### 3.4 Productivity at patch scale per species

**FIGURE 5:**
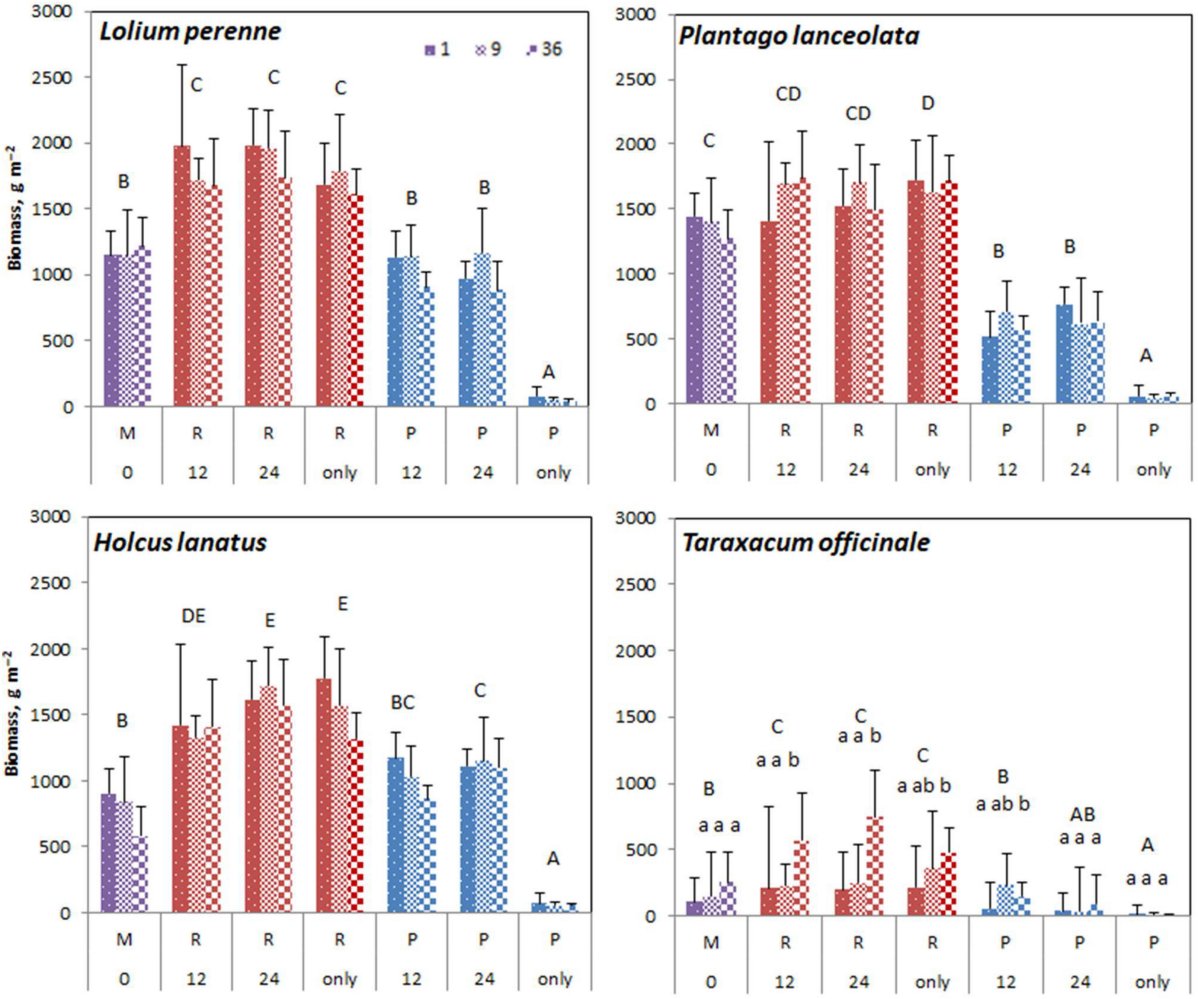
Biomass productivity ± SE from the first harvest of the four grassland species growing in mesocosms in function of soil patch type within the SH treatments for different clumping levels (1,9,36). On 12 and 24 heterogeneity mesocosms the data from the plants growing on the rich (R) and the poor (P) soil cells are shown separately. M denotes mixed soil. Significant differences are indicated with capitals among the soil heterogeneity treatments, and with small letters among the clumping levels within soil heterogeneity treatments.

In further analysis, we focused on the first growing period in which most of the seasonal growth occurred. By analyzing this biomass data per species and soil type in the patches of the uppermost layer, more patterns were discernable. Overall, the productivity of three species (*L. perenne*, *H. lanatus*, and *P. lanceolata*) was relatively similar with *L. perenne* having the highest productivity, whereas *T. officinale* was much less productive (Figure 5; Table S1).

Soil heterogeneity affected the biomass of all four species (Figure 5; Table S1). As expected, productivity was highest on the pure rich soil and the nutrient‐rich patches and lowest on the pure poor soil and its patches. Plants on a rich patch surrounded by poor patches grew as well as those growing on pure rich soil (Figure 5). However, plants on poor patches performed better when they had access to neighboring patches of richer soil than those growing on a completely poor mesocosm, except for *T. officinale* on the 24 cm poor patches.

The relative sensitivity of species to the soil patch quality was different. Although rich patches were always more productive than the poor patches of equivalent cell size, *L. perenne* and especially *H. lanatus* still grew relatively well on poor soil cells whereas *P. lanceolata* and *T. officinale* showed larger differences in growth between poor and rich patches of 12 and 24 cm.

Furthermore, there were some interesting differences in how species performed on the rich or poor patches compared to the mixed soil (M). All species except *P. lanceolata* grew significantly better on the 12 and 24 cm‐rich patches than on the mixed soil. In the poor patches, however, *H. lanatus* grew even better on the 24 cm poor cells than on mixed soil and *L. perenne* was as productive on poor patches as on mixed soil. *P. lanceolata* on the other hand grew significantly worse on poor patches than on mixed soil. Finally, the growth of *T. officinale* on the poor patches was comparable to that of mixed soil with a tendency to grow worse on larger patches.

Clumping affected the productivity of *H. lanatus* and *T. officinale* but in a contrasting way. *H. lanatus* grew better when planted alone than in clumps of 36, while *T. officinale* grew better in clumps of 36 than alone and clumps of 9. In *T. officinale*, the effect of clumping was modulated by the type of soil. Plants benefited from larger clumps only on rich soil patches. On poor patches of cell size 12 cm, it was clump size 9 that benefited productivity relative to growing alone while on larger poor patches and mixed soil, clumping had no effect.

### 3.5 PAR interception at patch scale

**FIGURE 6:**
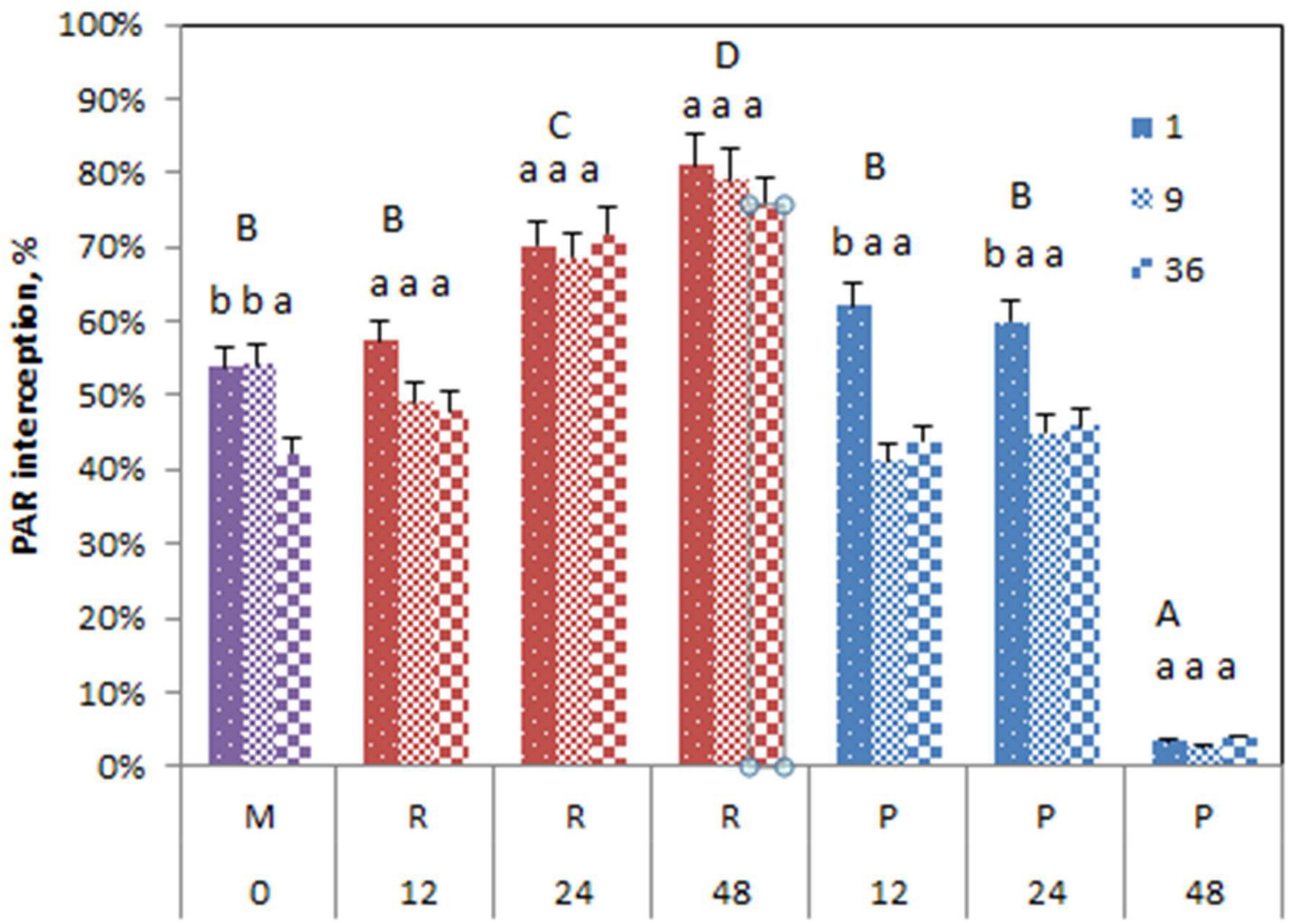
PAR interception (%) ± SE above rich (R), poor (P), or mixed soil (M) of different soil cell sizes, at three plant clumping levels (1,9,36 individuals). Significant differences are indicated with capitals among the soil heterogeneity treatments and with small letters among the clumping levels within soil heterogeneity treatments. See Figure 5 for treatment codes.

The vegetation in July 2017 was quite dense and PAR interception was >40% for all mesocosms except the pure poor treatments (Figure 6). Soil cell size affected PAR interception on rich soil patches, with the highest PAR interception found on the pure rich treatments followed by the rich 24 cm cell size and with the rest of treatments being comparable except pure poor soil which was lowest. In contrast to the biomass data, there were no differences in PAR interception between the rich and poor patches at 12 cm cell size, and the interception in R12 was considerably reduced compared to the bigger cell size.

In contrast to the results from the mesocosm scale, clumping as well as the interaction between clumping and treatment also significantly affected PAR interception (Table S1). Clumping at 9 or 36 plants overall decreased PAR interception relative to the evenly mixed treatments. This was especially true for treatments P12, P24, and M, yet in the latter only for the clumping of 36 plants (Figure 6).

Looking at the data over specific soil patches, the differences between rich and poor patches concerning light interception were much smaller than the differences in biomass.

### 3.6 Chlorophyll and NBI at patch scale per species

**FIGURE 7:**
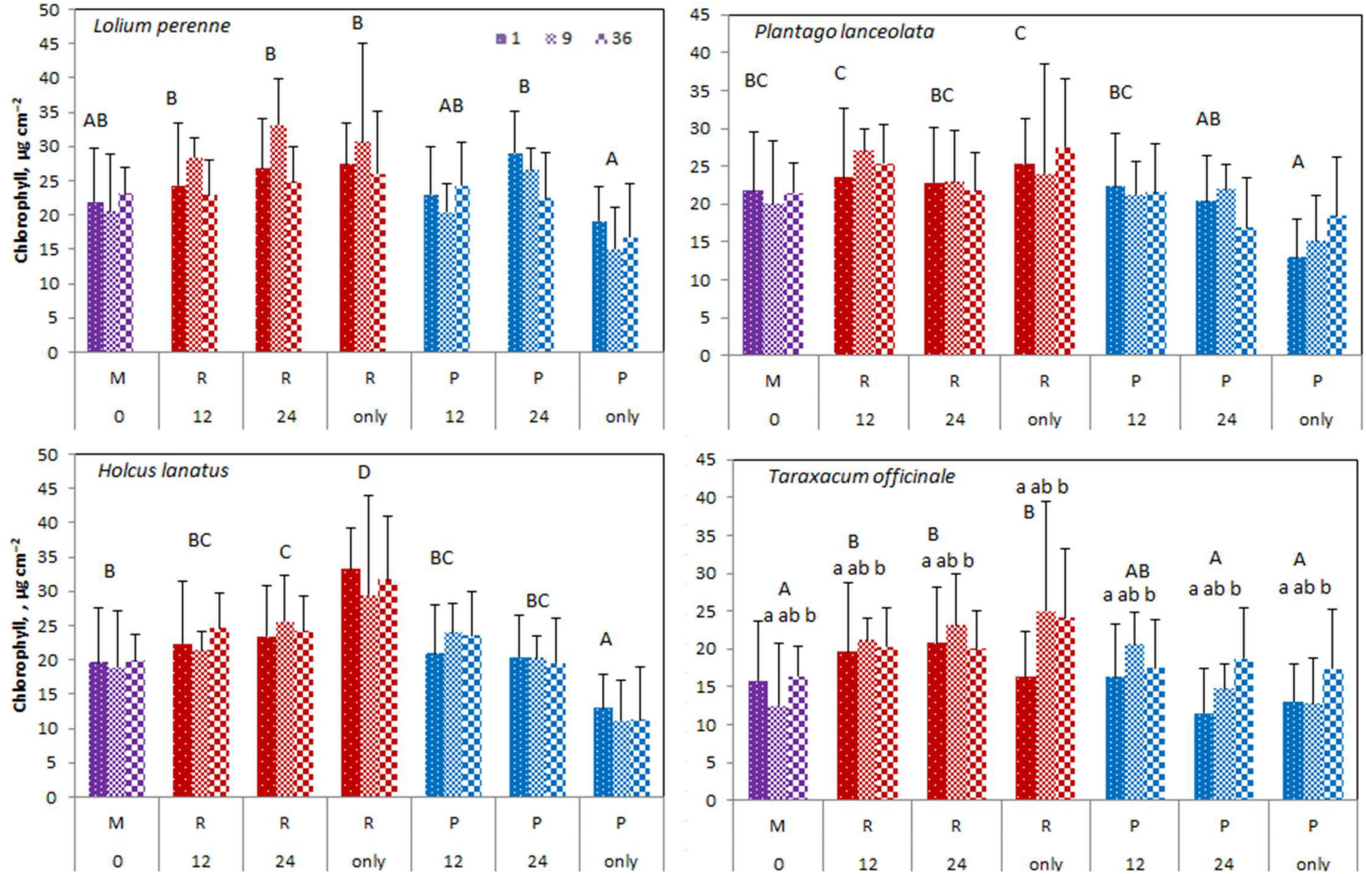
Chlorophyll content ± SE of the four grassland species growing in mesocosms in function of soil patch type within the SH treatments for different clumping levels (1,9,36). On 12 and 24 cm heterogeneity mesocosms the data from the plants growing on the rich (R) and the poor (P) soil cells are shown separately. Significant differences are indicated with capitals among the soil heterogeneity treatments and with small letters among the clumping levels within soil heterogeneity treatments. See Figure 5 for treatment codes.

In general, chlorophyll showed similar but less pronounced patterns compared to biomass productivity (Table S1), with the highest contents found in *L. perenne* (mean 24.1 μg/cm^2^), comparable contents in *H. lanatus* (21.8) and *P. lanceolata* (21.7) and lowest in *T. officinale* (18.0). Chlorophyll content was also impacted by soil heterogeneity (main effect) and this effect was dependent on the plant species (interaction effect; Table S1). For all species (Table S1; Figure 7), chlorophyll content was the highest on pure rich soil and lowest on pure poor soil with mixed soil yielding intermediate values, which were however significantly higher than those on pure poor soil for *H. lanatus* and *P. lanceolata* but were significantly lower than those on pure rich soil except for *L. perenne* and *P. lanceolata*.

In treatments with cell size 12 and 24 cm, there was a tendency for the rich soil patches to have higher chlorophyll content than the poor patches, but these differences were less pronounced than for productivity and statistically significant only for *T. officinale* on the 24 cm cell size. As for soil heterogeneity, soil cell sizes of 12 and 24 cm within one soil type did not differ in chlorophyll contents. Poor 12 and 24 cm patches had similar chlorophyll content as mixed soil and the R12 and R24 had higher chlorophyll content than the mixed soil only in *T. officinale*.

Clumping again affected *T. officinale* with the content for single plants lower than for those in clumps of 36. On the other hand, in *P. lanceolata*, no significant effects similar to those for biomass were found. Clumping did not affect any of the two grasses.

By the end of the season, all chlorophyll contents were relatively low which is normal at that phenological stage and need not indicate a nutrient shortage (data not shown).

The NBI followed similar patterns as chlorophyll (Figure S1) with some noteworthy exceptions. NBI was again highest in *L. perenne* and lowest in *T. officinale*, but this time similarly low also in *P. lanceolata*. Soil heterogeneity had again an effect on NBI. Mixed soil yielded higher values than pure poor soil this time only in *L. perenne* but was consistently lower than pure rich soil in all species.

In treatments of 12‐ and 24‐cell size, there were again no significant differences between the rich and poor patches, not even in *T. officinale*. In line with the observation on pure poor and rich soil and mixed soil, plants on poor patches (P12, P24) had similar NBIs to those on mixed soil. Plants in rich patches had higher NBIs than in the mixed soil this time in *H. lanatus* (R12, R24).

Clumping had an overall significant effect on the NBI of all species considered together (Table S1; Figure S2), with plants in clumps of 36 showing lower NBI than plants growing alone. This difference was mostly driven by *T. officinale* and partly also by *L. perenne* which showed a similar tendency (Figure S1). Interestingly, this trend was opposite to the pattern observed for chlorophyll.

### 3.7 Mortality

Mortality over the whole experiment period differed among species and also among soil heterogeneity treatments (Table S1). These patterns sometimes resembled those of productivity but not always. *T. officinale* had the highest mortality (mean 14.1%), followed by *L. perenne* (7.5%); the lowest mortality was recorded in *H. lanatus* (3.2%) and *P. lanceolata* (3.9%).

Across all species combined, the soil heterogeneity effect on mortality (Figure S3) was such that the highest mortality occurred on poor patches of cell size 24, which was higher than mortality on pure poor soil. Also interestingly, mortality on pure rich soil was second highest, significantly higher than R12 patches, where mortality was the lowest.

**FIGURE 8:**
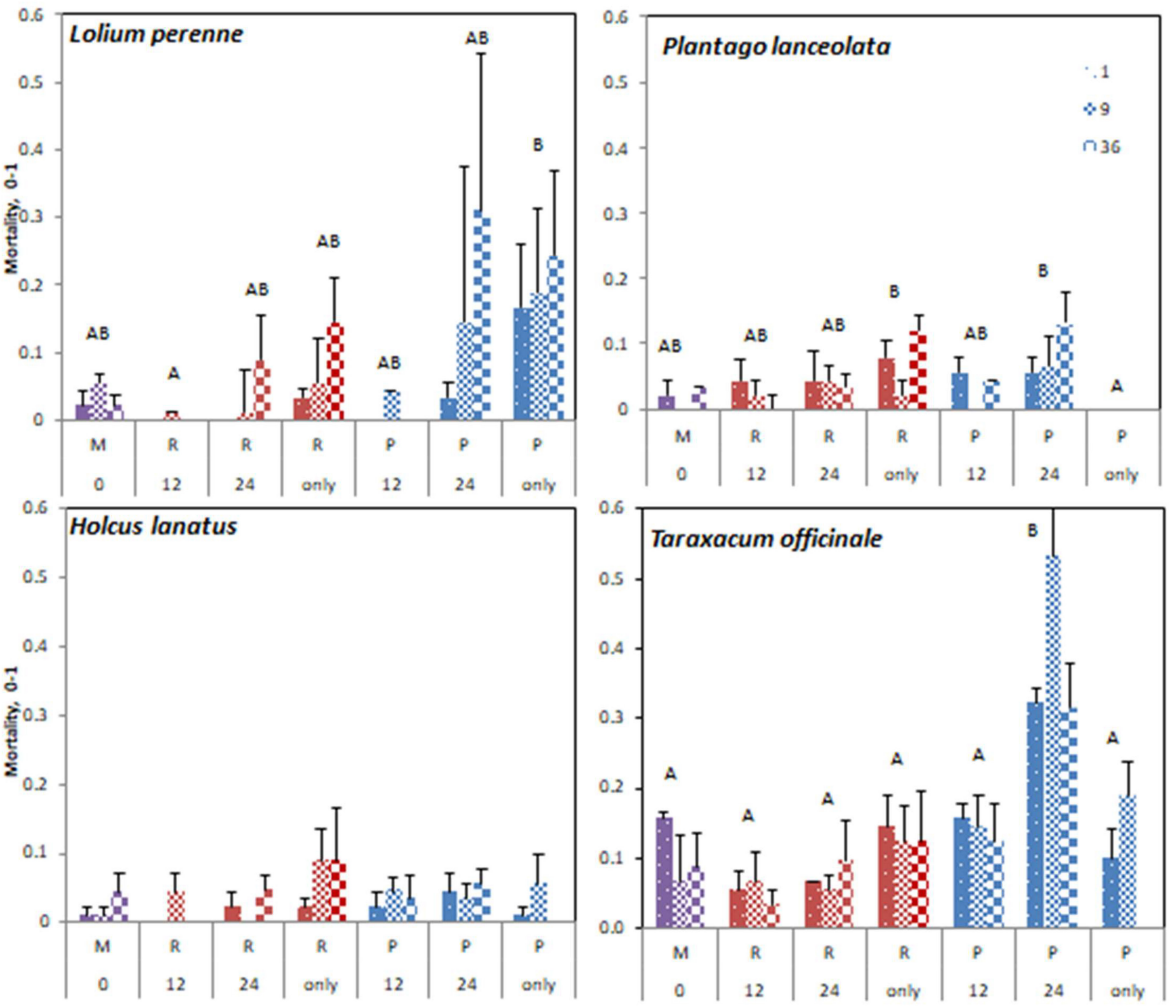
Mortality ± SE as fraction of individual plants lost in function of species, soil type, clumping, and soil heterogeneity treatment. See Figure 5 for treatment codes.

The effect of soil heterogeneity differed among plant species (Figure 8). In *L. perenne*, the highest mortality occurred on pure P soil and lowest on R12 patches. In *P. lanceolata*, the mortality was highest in pure R soil and P24 soil and the lowest in pure P soil. In *T. officinale* only the P24 patches stood out with the highest mortalities (39%) with other treatments ranging between 5% and 14% but not significantly differing from each other. Mortality of *H. lanatus* did not differ among treatments.

There was no significant effect of clumping on mortality.

## 4 DISCUSSION

### 4.1 Mesocosm scale

As hypothesized, soil heterogeneity significantly affected total aboveground productivity. Productivity was increased at intermediate soil cell sizes (12 and 24 cm) by about 20% compared to the 0 cm (mixed) or the average of the pure rich and poor mesocosms, resulting in an overall increased productivity at this soil heterogeneity. This is in line with the unimodal trend found in plant diversity (with a peak at 12 cm) in a previous study where a seed rain was applied to similarly constructed mesocosms (Liu et al., 2019). Intermediate soil cell sizes had the highest productivity likely because three niches were available to the plants: the center of the resource‐poor patches, the edge between patches and the center of the resource‐rich patches. From the niche perspective, the mixed soil provided only one niche because the patch size was too small (<1 cm) to represent separate niches and the 48 cm cells represented only two niches with no micro‐edge niche.

Stover and Henry (2018) and (2020b) hypothesized that micro‐edges have higher productivity and N retention due to complementarity, so the highest productivity would be found when there are distinct niches inside a patch compared to along the edges. This could explain our highest productivity at the 24 cm soil cell size at the second harvest because at 12 cm more of the surface can be seen as an edge which reduced the distinct niches inside the patches.

At soil cell size 48 cm, the growth on the poor soil was extremely low because plants could not get nutrients from neighboring patches or patches below. It is important to note that from our results it cannot be proven whether productivity would also be lower at 48‐cm soil cells in contact with each other.

The effect of soil heterogeneity was likewise found on PAR interception and nutrient uptake (chlorophyll content and NBI). PAR interception was highest on soil cell size 24 cm: the intermediate soil heterogeneity level thus maximized the capacity of the plant communities to capture the resource light. Although chlorophyll and NBI content was only measured on a leaf area basis and showed no significant changes, this implies an overall increase in nutrient uptake since leaf biomass and area (as approximated by interception) were increased.

Concerning water uptake, Liu, De Boeck, et al. (2017) using a similar setup of soil heterogeneity (but other species) analyzed root growth which could help interpret our results. They found no effect of soil cell size on root biomass, but a reduced root‐to‐shoot ratio on more heterogeneous soil and reduced root biomass in the deepest soil layers for plants growing on smaller soil patches (0–24 cm). They concluded that plants that were forced to forage more than on average 12 cm toward a patch of nutrient‐rich substrate exceeded a cost/benefit threshold. Our results suggest the same could have happened in our experiment: reduced root growth to the deepest layers for soil cell sizes 0–24 cm and possibly a reduced R/S ratio for plants on soil cell sizes 12 and 24 cm that had enough water and nutrients at a shorter distance would explain the pattern we see in water uptake.

In contrast to the major effects of soil cell size, average productivity of the mesocosms was unaffected by clumping, and no significant overall positive or negative interaction between soil heterogeneity and species clumping was found. Intraspecific aggregation or clumping has been shown to influence competitive interactions in experimental plant communities (Stoll & Prati, 2001) but such results depend on the chosen species as well as the level of competition. A similar lack of overall response to clumping was found concerning nutrient uptake except for a small reduction in NBI on the largest clumps possibly indicating that at this clump size, the soil in the center of the clump is not always fully exploited.

This reduction is in line with the theoretical concept of De Boeck et al. (2006) who hypothesized that clumping would reduce resource uptake because of reduced complementarity of the root systems within a clump. However, our clumps were small so this reduced uptake in the center of poor patches with the species clumped on it, appears to have been limited. In addition, overall nutrient (and water) availability in our mesocosms was high. The interaction between soil heterogeneity and clumping was limited to responses at the species level (see below).

Mortality was highest on the most productive mesocosms, which was probably caused more by competition for light than by lack of resources since chlorophyll and NBI levels were always high. This was expected since average nutrient levels were high and the mesocosms were watered to maintain sufficient water levels.

### 4.2 Impact of soil patch type

To improve our understanding of the mechanisms behind the enhanced productivity at intermediate soil cell sizes, we compared the rich and poor soil cells. Plants on a rich patch surrounded by poor patches grew as well as those growing on pure rich soil, while plants on poor patches performed better when they had access to neighboring patches of richer soil.

Two hypotheses could explain this result:

Hypothesis 1 The plants growing on rich patches surrounded by poor patches did not suffer from plants on the poor patches competing for nutrients. On the rich soil, plant growth was limited by other factors than soil nutrition.

Hypothesis 2 On the rich patches the plants did suffer from the increased competition for nutrients from plants on neighboring poor patches, but at the same time encountered less competition for light as these adjacent poor patches have sparser vegetation, and these two effects canceled each other out.

Chlorophyll content and NBI showed that for all species the soil cell type had a limited effect on the availability of nutrients. Hypothesis 2 seems marginally more likely because the highest NBI was found on the pure rich patches, indicating that under other circumstances nutrient levels were below optimal.

Soil cell size of 12 cm still showed large differences between the poor versus rich soil cells on growth (Figure 5) which shows there were still distinct niches, though the higher proportion of “edge” could explain the reduced productivity compared to the cell size 24 mesocosms. The effect of specific patch size would in other contexts of course depend on plant size.

The results on PAR interception clearly show that the plants growing on the rich patches encroached on the poor patches above the ground. On the pure rich mesocosms, the plants could not find free space and PAR interception was the highest, and on the pure poor mesocosms, PAR interception was very low. At 12 cm there was no difference between the poor and the rich patches, and the aboveground vegetation appeared uniform, while at 24 cm there was a difference between the denser rich and less dense poor patches. This indicates that at a patch size 12 cm, plants growing in neighboring (rich) patches can exploit the space above poor patches for light while intercepting less light above their own (rich) patches.

It is more difficult to explain the similar nutrient uptake on a larger poor patch (24 cm) compared to 12 cm since the distance to a high nutrient patch is greater, but if more C is allocated to the roots due to decreased aboveground competition, this could outweigh the disadvantage of the longer distance. Maestre and Reynolds (2007) reported a negative relationship between individual biomass and distance to a nutrient patch but in our setup apparently, the patches were never too distant for the plants to reach.

Mortality was higher in the poor patches, though this was only significant for *L. perenne* and *T. officinale*. Day et al. (2003) found that although plants on poor patches grow less, they also show less mortality because competition is reduced, resulting in an overall reduced mortality under heterogeneous soil conditions. In our experiment, we ascribe the mortality on the poor patches of intermediate size to intense aboveground competition in combination with reduced nutrient availability.

### 4.3 Species‐specific responses

Both the effects of soil heterogeneity and clumping were species‐specific. The mass ratio hypothesis suggests that the ecosystem response is determined by the functional identity of the dominant species (Mokany et al., 2008). In our experiment, three of the four species were co‐dominant (*L. perenne*, *H. lanatus*, and *P. lanceolata*), and we can link the effects of soil heterogeneity on productivity to the two co‐dominant grass species. Both two dominant grass species (*L. perenne* and *H. lanatus*), as well as the suppressed species (*T. officinale*), followed the observed unimodal trend in productivity in response to soil heterogeneity.

*P. lanceolata* on the other hand did not follow this trend and showed the highest biomass at soil cell size 0 (mixed soil) possibly indicating that the growth of *P. lanceolata* in mixed soil was not nutrient‐limited. The lower productivity of *P. lanceolata* at larger cell sizes was related to a strong reduction of productivity in the poor soil cells, indicating that its ability to exploit other patches was lower than in the grasses, whereas the productivity was unchanged in the rich cells (Figure 5; Table 2). These results suggest *P. lanceolata* was not a strong competitor for nutrients, possibly due to low root plasticity so plants on the poor patches were not able to compete for the nutrients of neighboring rich patches. *P. lanceolata* had a low NBI but an average chlorophyll content and this could indicate the species did not suffer from deficiency. Clumping did not significantly impact the performance of *P. lanceolata*.

Soil heterogeneity had a significant effect on the productivity of *L. perenne*, with the highest biomass found at 12 and 24 cm soil cell size. This competitive species underwent more intra‐ versus inter‐species competition resulting in a significant effect of clumping where growth was reduced on rich soil when the plant was grown in clumps of 9 or 36. Comparing the growth on the rich and poor patches it is clear that *L. perenne* grows almost as well on a poor patch compared to a mixed patch (with the exception of 48 cm poor mesocosm where there is no neighboring rich patch). The pattern is very similar to the one of *H. lanatus* indicating both species can fully benefit from rooting into neighboring rich patches. However, the difference in growth between poor and rich soil cells was greater in *L. perenne*. This suggests *Lolium* is mainly a strong competitor for light while *Holcus* is more efficient in exploring soil with intermediate‐size patches. Although *H. lanatus* was as productive as *L. perenne*, there are a few important differences. At 24 cm, the *H. lanatus* plants on the rich patches grew better than on the 12 cm rich patches, while on the poor patches, there was no significant difference in productivity, which explains the peak in total productivity at 24 cm (Figure 5). This confirms what was found by Maestre and Reynolds (2007) that *H. lanatus* manages very well to explore the soil in neighboring patches. The chlorophyll content of *H. lanatus* was as high on 12 cm poor patches as on 12 cm rich patches (Figure 6), which confirms this hypothesis. *H. lanatus* is less competitive on mixed soil with cell size 0, where it loses its competitive advantage of root proliferation.

*T. officinale* showed the same response to soil heterogeneity as the dominant grasses, with the highest values at 12 and 24 cm. Its poor competitiveness was likely due to the low cutting frequency and heavy shading by the grasses as increasing grass height has been shown to decrease *T. officinale* density, at least partly due to shading (Mølgaard, 1977). Possibly root morphology played a role as well, as the taproot is not suited to proliferate laterally in neighboring patches of better soil. However, chlorophyll and NBI content of *T. officinale* growing on the poor soil cells was not significantly lower, possibly because the taproot can reach the deeper soil and in our 3D soil under a poor patch there was always a rich patch. The tendency to lower biomass and chlorophyll on the poor 24 cm patches (compared to the 12 cm patches) could indicate it can benefit from neighboring rich patches only to a limited distance of less than 24 cm.

Clumping increased the growth of *T. officinale* as expected since an inferior competitor is more protected within a clump, although the average mortality (% dead individuals) was not significantly affected by clumping or soil cell size. This also shows that the mortality of the species was probably more due to shading (total productivity of these 24‐cm mesocosms was highest) than to nutrient limitation, and the shading of the tall grasses was high also above the clumps of *T. officinale*.

### 4.4 Effect of soil heterogeneity and clumping across scales

Our results on the effect of soil heterogeneity on productivity and species coexistence are also a confirmation of the hypothesis of Tamme et al. (2010) that small‐scale soil heterogeneity is a niche that can change the competitiveness of species but does not in itself increase diversity. This is also found in our study where *P. lanceolata* was competitively more advantaged on a mixed soil because at greater soil cell sizes it is less able to proliferate its roots into adjacent patches.

Xue et al. (2018) also found that soil heterogeneity can promote the coexistence of species by reducing the growth inequality of two competing species, even though the same species in monoculture are more productive on mixed soil. As hypothesized, clumping had a positive effect on the inferior species. Zhang et al. (2014) found that increasing the ratio of interspecific/conspecific interactions between species (decreased clumping) increases ecosystem productivity. In their experiment, the dominant plants grew much better, which outweighed the suppressed species that remained smaller. However, we did not find a significant effect on clump size at the mesocosm scale. In our study, the clump sizes were small compared to the size of the dominant grasses, so these were growing over the entire mesocosms aboveground (largest clump size was 36 individual plants or 24 cm). In addition, nutrient levels were quite high, so even on the poor patches, growth was barely reduced. This is probably why the clumping effects were minimal.

In summary, our results suggest clumping can play a role in maintaining diversity by supporting inferior species without affecting productivity.

### 4.5 Limitations

Although our experiment produced a unique dataset on the combination of controlled 3‐dimensional soil heterogeneity and species clumping, the conclusions should be handled with caution as the setting and specifically the species interactions are artificial and the mesocosms were newly planted (for example, it is unclear how these mesocosms would evolve in the longer term). Moreover, potentially important interactions with belowground microbial diversity and community composition, which were not studied, might also explain some of our results. For example, Hendriks et al. (2015) showed that spatial heterogeneity can increase productivity due to the reduction of negative plant–soil feedback. The impact of soil fauna may also play a role, as recorded by Stover and Henry (2020a) who found that increased soil heterogeneity promoted soil macrofauna, which would also affect ecosystem processes.

### 4.6 Management implications

Overall, the knowledge gained from our results may be important for interpreting experimental and field data and may provide a basis for improving management practices. For example, in grasslands, clumped sowing might improve diversity and coexistence which could improve ecosystem resistance/resilience to climate extremes (Oliveira et al., 2022), without reducing productivity. However, Liu et al. (2021) found small‐scale soil heterogeneity can increase drought stress because of the enhanced biomass production.

Heterogeneous fertilization could perhaps allow for a reduction in fertilizer use. Whether management can effectively be improved by putting heterogeneity to use remains an open question, but understanding the role of soil heterogeneity is clearly crucial to answering it. Also, we found that a dominant grass species that can exploit the complete range of soil conditions of the different patches can outcompete others, which confirms the studies of Baer et al. (2005), Xue et al. (2018) and Yang et al. (2015), indicating that the level of soil nutrients should also receive proper attention in possible applications of soil heterogeneity.

## 5 CONCLUSIONS

In our experiment, we manipulated both soil and plant spatial heterogeneity to investigate their isolated and combined effects on plant productivity and resource uptake.

We found that soil heterogeneity had a significant effect on aboveground productivity. Whole‐mesocosm aboveground productivity was higher on soil of intermediate heterogeneity (soil cell sizes 12–24 cm) by more than 20%, mainly through increased plant growth on poor soil cells. The overall resource uptake of these mesocosms (soil cell sizes 12–24 cm) was higher for nutrients and light, but not for water. Not all species showed the highest productivity on these intermediate soil cell sizes, but the dominant grasses (*Lolium perenne* and *Holcus lanatus*) and the inferior species (*Taraxacum officinale*) did. In contrast, *Plantago lanceolata* was most productive at soil cell size 0 likely because of its limited ability to explore neighboring rich soil patches when grown on poor soil.

Species clumping did not significantly affect total mesocosm productivity though there were some significant effects at the species level, both individually, or in interaction with soil heterogeneity. Clumping increased the growth of the inferior species *Taraxacum officinale* while the growth of one dominant grass species, *Holcus lanatus*, was reduced. The interaction between clumping and soil heterogeneity was found only for the productivity of *T. officinale* which benefited from the largest clumps at rich soil patches and from intermediate clumping (by nine individuals) at the smallest (12‐cm) poor soil patches and was not affected by clumping in larger poor soil patches and mixed soil.

Overall, our results show that productivity is highest at intermediate soil heterogeneity and that clumping can improve the growth of inferior species without affecting overall productivity.

## AUTHOR CONTRIBUTIONS

**O. Vindušková:** Data curation (supporting); formal analysis (equal); software (lead); visualization (equal); writing – original draft (equal); writing – review and editing (lead). **G. Deckmyn:** Formal analysis (equal); visualization (equal); writing – original draft (lead); writing – review and editing (equal). **M. Bortier:** Investigation (lead); writing – original draft (equal). **H. J. De Boeck:** Conceptualization (supporting); supervision (equal); writing – original draft (supporting); writing – review and editing (supporting). **Y. Liu:** Investigation (supporting); writing – original draft (supporting); writing – review and editing (supporting). **I. Nijs:** Conceptualization (equal); funding acquisition (equal); methodology (equal); supervision (equal).

## Supporting information


Appendix S1

## Data Availability

The data that support the findings of this study are openly available in Dryad at https://doi.org/10.5061/dryad.7wm37pw00.
