# Supplementary material for: Combined effects of soil 3D spatial heterogeneity and biotic spatial heterogeneity (plant clumping) on ecosystem processes in grasslands
Source: Ecol Evol. 2023 Oct 24;13(10):e10604. doi: 10.1002/ece3.10604 (PMC10597742; doi:10.1002/ece3.10604)
Supplement: Supplementary file 1 — Appendix S1 [file ECE3-13-e10604-s001.docx]

# Appendix

**Table S1**. Results of the linear mixed effect model analyses on patch and species level data. In these analyses, soil heterogeneity factor included soil patch types within treatments and had 7 levels (M, R12, R24, pure R, P12, P24, pure R; see Fig. 5 for treatment codes). Asterisks denote p-values < 0.001 (***), 0.01 (**) and 0.05 (*).
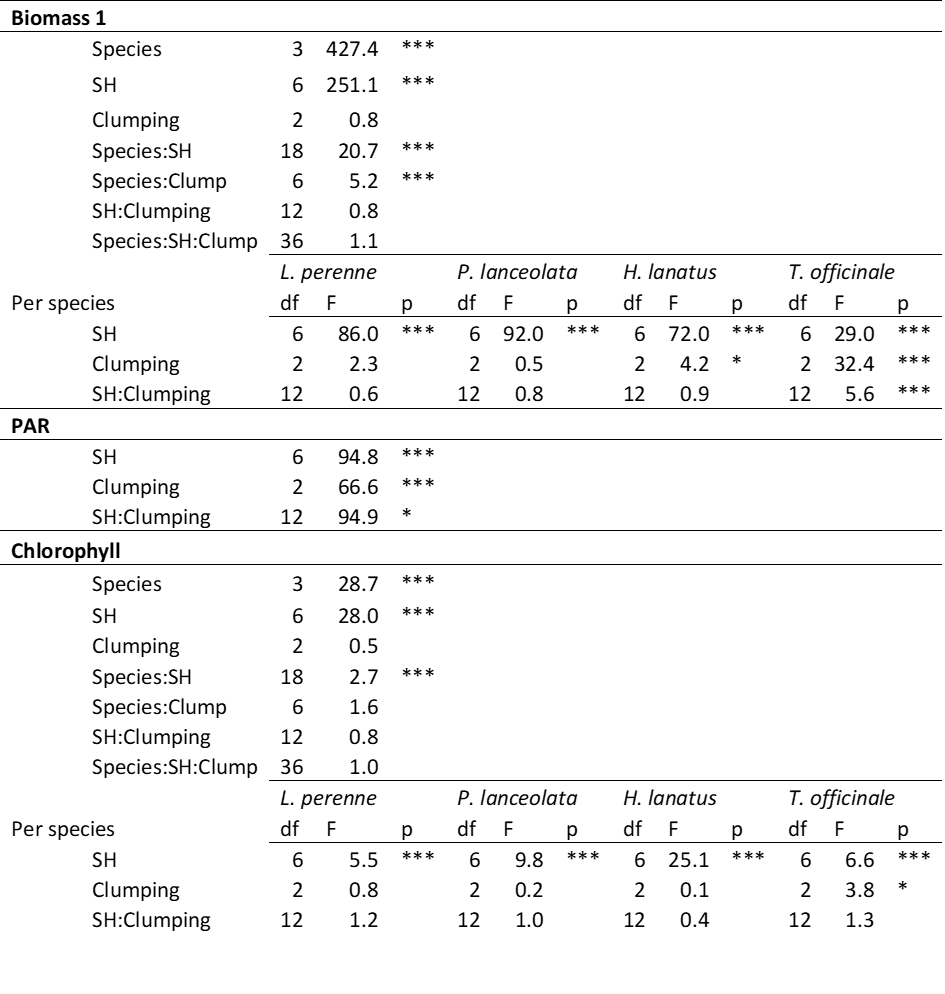


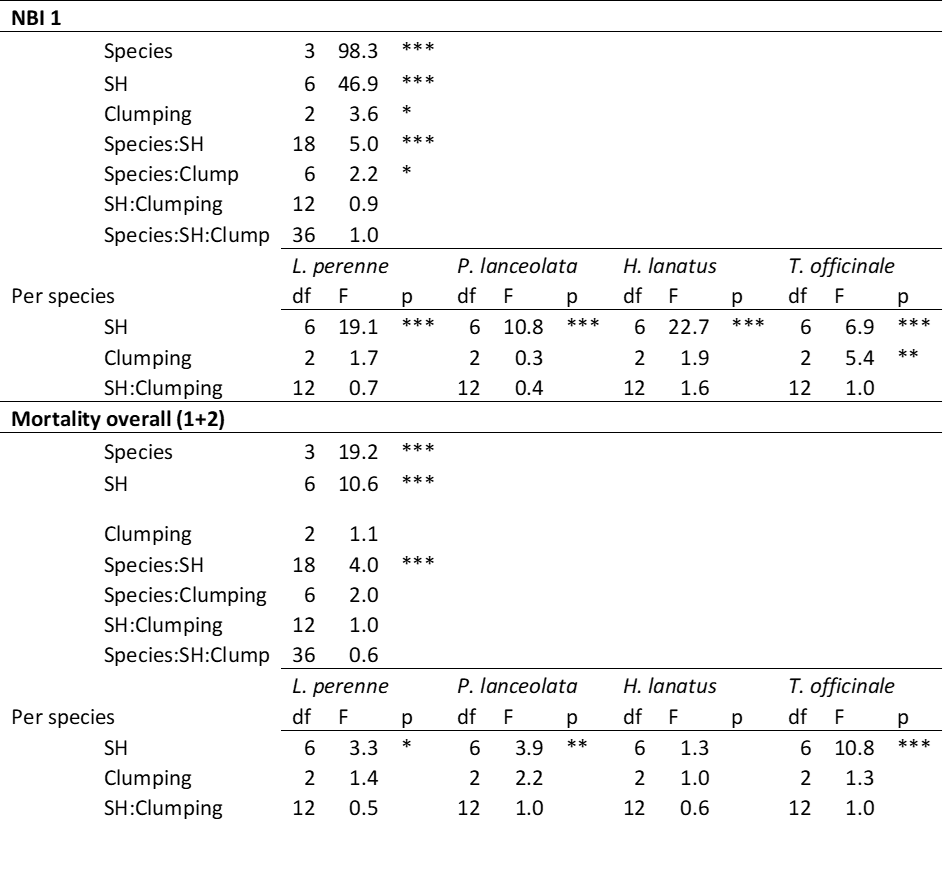


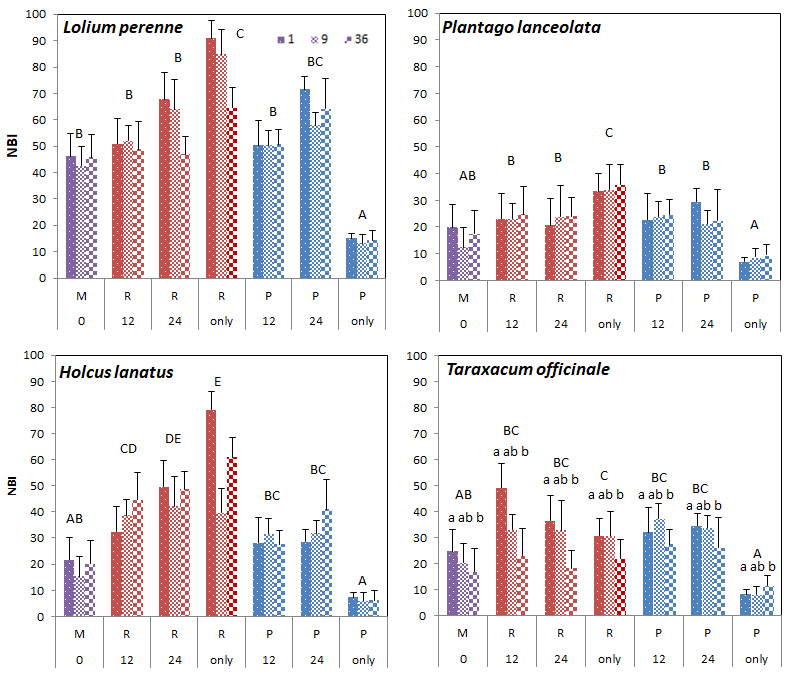


***Fig. S1.*** *Nitrogen balance index NBI ± SE of the different species in function of soil heterogeneity treatment (see Fig. 5 for codes). Significant differences between the treatments are indicated with capitals.*


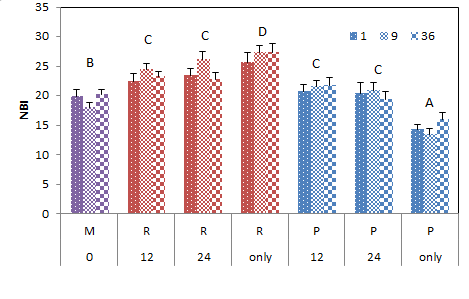


***Fig. S2.*** *Nitrogen balance index NBI ± SE of mesocosms in function of soil heterogeneity treatment (see Fig. 5 for codes) for different clumping levels (1,9,36). Significant differences between the treatments are indicated with capitals.*


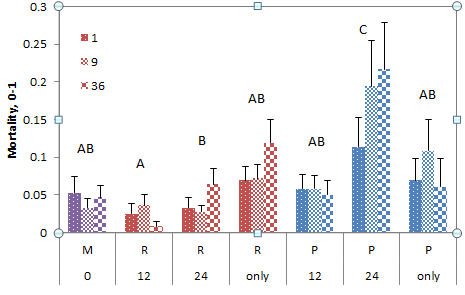


***Fig. S3.*** *Mortality (as fraction of individual plants) ± SE of mesocosms in function of soil heterogeneity treatment (see Fig. 5 for treatment codes) for different clumping levels (1,9,36). Significant differences between the treatments are indicated with capitals.*
